# Harnessing the benefits of seed oils: a comprehensive study on their role in functional foods

**DOI:** 10.1186/s13568-025-01875-9

**Published:** 2025-05-26

**Authors:** Marwa A. Kamel, Amira A. Gamal, Sayeda A. Abdelhamid, Marwa M. El-Said, Tamer M. El-Messery, Hamdy A. Zahran

**Affiliations:** 1 Environmental Virology Lab, Water Pollution Research Department, Environment and Climate Change Research Institute, National Research Centre, Dokki, Giza, 12622 Egypt; 2 Chemistry of Natural and Microbial Products Department, Pharmaceutical and Drug Industries Research Institute, National Research Centre, Dokki, Giza, 12622 Egypt; 3 Microbial Biotechnology Department, Biotechnology Research Institute, National Research Centre, Dokki, Giza, 12622 Egypt; 4 Dairy Department, Food Industries and Nutrition Research Institute, National Research Centre, Dokki, Giza, 12622 Egypt; 5 Fats and Oils Department, Food Industries and Nutrition Research Institute, National Research Centre, Dokki, Giza, 12622 Egypt

**Keywords:** Antioxidant activity, Antimicrobial, Anticoagulant, Fixed oils, Prebiotic, Health benefits

## Abstract

There has been a growing interest in functional foods in recent years to improve health and boost immunity, particularly since the COVID-19 pandemic, which reflects their significant role in promoting health and preventing various diseases, especially metabolic disorders. This study investigated the antimicrobial, antioxidant, anticoagulant, and prebiotic activities of six different oils: *Calotropis procera* oil (CPO), Chia seed oil (CSO), Moringa oil (MO), Neem oil (NO), Black seed oil (BSO), and Wheat germ oil (WGO) and their potential applications in health and nutrition. The DPPH and ABTS assays were used to evaluate the antioxidant activity of these oils. A good diffusion assay and minimum inhibitory concentrations (MIC) method were used to investigate the antimicrobial activity against pathogenic bacteria and fungi of human interest. Also, the prebiotic activities of oils were tested on three probiotic strains of *Lactobacillus* to evaluate their role in promoting the growth of beneficial bacteria against the pathogenic *E. coli.* Furthermore, the haematological effect of these oils was investigated in vitro through measuring their anticoagulant, and Fibrinolytic activity. The results demonstrated that DPPH assay revealed that CPO and WGO exhibited the highest antioxidant activity with IC50 values of 15.2 µg/mL and 18.7 µg/mL, respectively, while BSO showed the least activity with an IC50 of 45.3 µg/mL. Antimicrobial activity, assessed using inhibition zone diameters, showed that CPO had the strongest effect against *Staphylococcus aureus* with a zone of 22 mm, followed by CSO at 19 mm. In terms of anticoagulant activity, CSO demonstrated the most potent fibrinolytic effect with a clot lysis percentage of 78%, while MO exhibited weaker activity at 35%. Prebiotic testing revealed that individual oils had limited effects on *Lactobacillus* growth, but a synergistic blend enhanced growth by 25% compared to controls. Overall, this study highlights the diverse health benefits of these oils and their potential as functional food ingredients that could contribute to improved health.

## 1. Introduction

The growing interest in functional foods reflects their significant role in promoting health and preventing various diseases, including metabolic disorders such as diabetes, gastrointestinal issues such as diarrhea and constipation, and even certain types of cancer (Thirumdas et al. 2021; Ponte et al. 2025; Elhadef et al. 2025; Mirzaei et al. 2025). These foods are recognized not only for their nutritional value but also for their ability to combat oxidative stress and inflammation through natural antioxidants, which can mitigate ageing processes and suppress mutagenesis and carcinogenesis (Kassem et al. 2017; El-Said et al. 2021; Monticolo et al., 2021; Ramadan et al. 2024a). Furthermore, functional foods enhance overall well-being by acting as bioregulators that improve appetite, nutrient absorption, immune response, and allergy suppression (Ramadan et al. 2024b; Pathak et al. 2025; Bhattarai et al. 2025).

Among these functional foods, lactic fermentation products such as yoghurt are particularly noteworthy due to their probiotic content (Ramadan et al. 2021; Abbas et al. 2024). Numerous studies highlight the critical role of probiotics in maintaining gut health and supporting the immune system (Ashaolu, 2020; Gohil et al., 2021; Liu et al. 2022). However, the over-prescription of antibiotics during the COVID-19 pandemic has exacerbated issues of antibiotic resistance and disrupted the gut microbiota (Lynch et al. 2020; Getahun et al. 2020; Sulayyim et al., 2020; Livermore et al., 2021; Ahmed et al., 2022; Chitungo et al. 2022). Pathogens such as *Escherichia coli* and Salmonella sp. have been implicated in gastroenteritis and foodborne outbreaks, with some strains exhibiting antibiotic resistance (Cutter, 2000; Callejón et al., 2015). This situation has intensified the search for natural substances with antimicrobial properties that can also provide antioxidant benefits, thereby addressing the challenges posed by antibiotic resistance and synthetic additives (Ketenoglu et al., 2020; Cheikhyoussef et al., 2020).

Plant seed oils have garnered increasing attention for their notable antioxidant and antimicrobial properties, which play a pivotal role in promoting human health. These oils are abundant in unsaturated fatty acids and bioactive compounds, endowing them with robust radical scavenging capabilities that effectively mitigate oxidative stress—a key factor implicated in the development of chronic diseases and the ageing process (Petropoulos et al. 2021; Mahmoud et al. 2015; Al-Rowaily et al. 2020a, b; Gebremeskal et al. 2024). Historically, these natural substances have been integral to traditional medicinal practices such as Ayurveda and Traditional Chinese Medicine, where they have been employed to address a wide spectrum of health conditions (Chopra et al. 1956; Nadkarni et al. 1976; Wealth of India 1985; Al-Jishi, 2000; Wang et al. 2018).

For instance, oils derived from seeds such as linseed, pumpkin, and *Nigella sativa* have demonstrated notable antimicrobial activity against various pathogens, making them valuable alternatives to synthetic preservatives in food products (Petropoulos et al. 2021; Bhavikatti et al., 2024). The presence of phenolic compounds and essential fatty acids in these oils not only enhances their antioxidant capacity but also supports immune function and reduces inflammation (Khémiri et al. 2020; Mettwally et al. 2022a, b; Mohammed et al. 2025). Furthermore, the incorporation of seed oils into diets can promote gut health by modulating the microbiota and providing prebiotic effects, thereby improving overall well-being. As research continues to unveil their health benefits, plant seed oils are poised to play a crucial role in the development of functional foods aimed at enhancing health and preventing disease.

In this study, we investigated six seed oils: Black seed (*Nigella sativa* L.) oil, Chia seed (*Salvia hispanica* L.) oil, Neem oil (*Azadirachta indica* L.), Wheat germ oil, Moringa (*Moringa oleifera* L.) oil, and *Calotropis procera* oil, along with a blend of these oils. The focus is on evaluating their antioxidant properties, antimicrobial efficacy, and prebiotic effects. The ultimate goal is to explore their potential for fortifying dairy products as appealing, functional foods suitable for both children and adults.

## Materials and methods

### Seeds oil extraction

The extraction of seed oils was performed using a hydraulic press, a method chosen for its efficiency and ability to preserve the quality and nutritional properties of the oils. Prior to extraction, the seeds were meticulously cleaned and dehulled to remove impurities and enhance oil yield. A sample of 100 g of seeds was placed in the hydraulic press chamber, where a pressure of 50–55 MPa was applied using a hydraulic cylinder. This high pressure ruptured the seed matrix, allowing the oil to be released into a collection tank while the seed cake was expelled. This solvent-free extraction process ensured that the oils retained their natural flavors and bioactive compounds due to minimal heat exposure (Naeem et al. 2019).

To standardize the oils for comparison, Gas Chromatography-Mass Spectrometry (GC-MS) was employed to profile their fatty acid composition. The oils were first subjected to transesterification, converting triglycerides into fatty acid methyl esters (FAMEs). These FAMEs were then analyzed using GC-MS to identify and quantify individual fatty acids based on established standards. This profiling ensured consistency in the composition of the oils used in subsequent experiments, enhancing the reliability and reproducibility of the study’s findings.”

### GC-MS profiling of oil fatty acids

GC-MS was used to examine the composition of fatty acids. Following a modified method by Alloh et al. (2024). A 10 mg sample was diluted in 2 mL of n-heptane, and then, 4 mL of 2 M methanolic KOH was added. The combination was subsequently centrifuged at 4000 rpm for 10 min, and the resultant n-heptane layer was obtained for gas chromatography (GC) examination. The study utilized an Agilent GC–MS/MS system (7890B GC-7010B MS, Gerstel, Germany), with a flame ionization detector (FID) and a capillary Agilent J&W DB-WAX column (60 m × 0.25 μm × 0.25 μm). The oven temperature was first set at 50 °C for 1 min, then raised to 200 °C at a rate of 25 °C per minute. Subsequent to 10 min, the temperature was elevated to 230 °C at a rate of 3 °C/min and sustained at this level for 18 min. The injector and detector temperatures were established at 250 °C and 300 °C, respectively. A 1 µL sample was injected at a carrier gas flow rate of 1 mL/min, with a split ratio of 1:40. Fatty acid identification was conducted by comparing retention periods with those of a FAME mix of 37 standard components (Supelco 37, Bellefonte, PA, USA).

### Microbial strains and media

Four bacterial strains, one fungal strain, and one yeast strain of significant importance were utilized to evaluate the antibacterial properties of the plant and seed oils. The bacterial strains included two Gram-positive species: *Staphylococcus aureus* NRRL B-313 and *Bacillus subtilis* NRRL B-94, as well as two Gram-negative species: *Escherichia coli* NRC B-3703 and *Pseudomonas aeruginosa* NRC B-32. Additionally, the fungal strain *Aspergillus niger* NRRL 599 and the yeast strain *Candida albicans* NRRL 477 were included in the study. Nutrient agar media was employed for the growth of bacterial strains, while malt extract agar (MEA), prepared according to Dutton and Penn (1989), was used for the fungal and yeast strains. The typical formulation for the MEA (g/L) consisted of 30.0 g malt extract, 5.0 g peptone, and 15.0 g agar. The liquid medium was sterilized by autoclaving at 121℃ for 20 min before being used for subculturing; subsequently, solid media were prepared for the agar-well diffusion assay.

For the antifungal assays, Mycostatin was used as a positive control at a concentration of 100,000 IU/ml for both *Aspergillus niger* and *Candida albicans*. A total of 200 µL of the antifungal agent was inoculated into a 3 ml nutrient broth culture and incubated in a shaking incubator at 150 rpm and 37 °C for 36 h. Microbial growth was assessed by measuring the optical density at 620 nm, with results expressed as a percentage of growth inhibition.

### Well diffusion assay

The well diffusion method was employed to assess the antimicrobial activity of various plant and seed oils, including a blended oil. In this procedure, 10 mL of agar medium (nutrient and malt) was poured into sterile Petri dishes, followed by the addition of 15 mL of a seeded medium that had been previously inoculated with a bacterial suspension to achieve a concentration of 10^5^ CFU/mL. The microorganisms were cultured and incubated at 37 °C for 24 h. To ensure a uniform distribution of bacteria, the inoculum suspension was spread evenly over the agar plates using a sterile spreader.

Next, a sterile borer was used to create wells with a diameter of 9 mm in the inoculated media. A total of 100 µL of each oil, prepared at a concentration of 100 mg/mL, was then added to the wells. The plates were refrigerated at 4 °C for 2 h. to allow for diffusion of the essential oils before being incubated at 37 °C for an additional 24 h. The presence of inhibition zones around the wells was measured as an indicator of antibacterial activity (Dutton and Penn 1989). The antibacterial efficacy of the tested oils was compared to that of a broad-spectrum antibiotic, Ampicillin, also applied at a concentration of 100 mg/mL (Gibco™, Thermo Fisher Scientific, USA), serving as a positive control against the bacterial strains. For antifungal activity, Mycostatin was used as a control at a concentration of 100,000 IU/ml.

### Determination of minimum inhibitory concentrations (MIC)

The minimum inhibitory concentration (MIC) is defined as the lowest concentration of an antimicrobial agent that inhibits microbial growth after twenty-four hours of incubation on agar plates. To determine the MIC of the six oils exhibiting strong antibacterial activity, as well as a mixture of these oils, the well diffusion method was employed. Using a sterile borer, wells with a diameter of 9 mm were created in the inoculated agar media, into which 100 mg/mL of each oil was added. The volumes tested varied by oil type: *Calotropis procera* oil was evaluated at volumes ranging from 10 to 100 µL; Chia seed oil from 15 to 30 µL; Moringa oil from 15 to 100 µL; Neem oil from 15 to 125 µL; and both Black seed and Wheat germ oils from 10 to 50 µL.

Agar plates were prepared by pouring the medium seeded with microbial suspensions of pathogenic strains. The plates were then refrigerated at 4 °C for 2 h. to allow for diffusion before being incubated at 37 °C for 24 to 48 h. Two biological replicates were conducted for each strain, and the MIC values were calculated for each replicate. These values were defined as the lowest tested concentration of oil extract at which ≤ 100% and ≤ 0.1% of bacterial growth remained after 24 h. This method provides a quantitative assessment of the antimicrobial effectiveness of the oils, contributing valuable insights into their potential therapeutic applications against bacterial infections.

### Oil mixture Preparation

To achieve the optimal antibacterial activity, a synergistic mixture of the selected plant and seed oils was formulated as follows: 5% Calotropis procera oil, 20% Chia seed oil, 20% Moringa oil, 5% Neem oil, 25% Black seed oil, and 25% Wheat germ oil. This combination leverages the unique antibacterial properties of each oil while maintaining a balanced profile to maximise overall effectiveness. To refine this mixture, preliminary testing should be conducted using the well diffusion method to assess the antibacterial activity of various combinations at different concentrations. A statistical analysis, such as a simplex-centroid design, can help identify optimal ratios that yield the highest efficacy against specific bacterial strains. Finally, validation through replicative testing will confirm the antimicrobial effectiveness of the optimized mixture, ensuring it is both potent and safe for potential therapeutic applications.

### Prebiotic activity of oils

` The prebiotic activity of the six oils, as well as a mixture of these oils, was evaluated experimentally. Three probiotic strains—*Lactobacillus casei*, *Lactobacillus reuteri*, and *Lactobacillus helveticus*—were cultured in MRS medium, while *Escherichia coli* was grown in nutrient broth, both at 37 °C for 24 h. Aliquots of 0.1 mL from each bacterial culture were used as inocula for 10 mL of the respective media (MRS and nutrient broth) supplemented with 100 µL of the oil samples being tested. After incubation at 37 °C for an additional 24 h., bacterial growth was measured at 625 nm using a blank of un-inoculated medium as a reference (Mettwally et al. 2022a, b). The prebiotic activity was quantified using the “Prebiotic Index” (I), calculated as follows: Prebiotic Index (I) = O.D. of bifidogenic bacterial culture / O.D. of pathogenic *E. coli* culture. All experiments were conducted in duplicates, and results are presented as means with standard deviations calculated for accuracy.

### Antioxidant activity of the oil

#### DPPH free-radical scavenging

The antioxidant activity of the six oils, as well as their mixture, was assessed using the DPPH method to evaluate the free radical scavenging activity of the sulfated polysaccharides, following the protocol outlined by Mettwally et al. (2022a, b) with slight modifications. In the first test tube, 1 mL of freshly prepared 2,2-diphenyl-1-picrylhydrazyl (DPPH) solution (0.1 mM) was combined with 100 µL of each oil sample. In the second tube, 1 mL of methanol was mixed with 100 µL of each oil sample to serve as a blank. The third tube contained 1 mL of DPPH solution mixed with 100 µL of methanol, serving as the control. Each tube was vortexed and allowed to incubate in the dark at 37 °C for one hour. The absorbance of each mixture was then measured using a spectrophotometer (Jenway 6715 UV/Visible) at 517 nm. The antioxidant capacity to scavenge the stable DPPH free radical was calculated using the following Eq. 1:

1 $$\:Scavenging\:Activity\:\left(\%\right)=\frac{Abs1-Abs2}{Abs1}\:x\:100$$

Where Abs₁ is the absorbance of the control, and Abs₂ is the absorbance of the sample.

#### Antioxidant activity by ABTS method

The ABTS radical-scavenging activity was determined according to the method of Asghari et al. (2018) with some modifications. Briefly, ABTS solution (7 mM) was mixed with potassium persulfate (2.45 mM) and kept in the dark at room temperature for 15 h to reach a stable oxidative level. Then, the solution was diluted with distilled water to an absorbance of 0.7 (± 0.02) at 734 nm. After adding 200 µL of ABTS solution to 50 µL of the tested oil, the absorbance was recorded using a spectrophotometer at 734 nm after 20 s. The percentage of ABTS radical inhibition was calculated according to the Eq. 2:

2 $$\:ABTS\:inhibition\:\left(\%\right)=\frac{Abs1-Abs2}{Abs1}\:x\:100$$

Where Abs₁ is the absorbance of the control, and Abs₂ is the absorbance of the sample.

### Anticoagulant activity of oil

The anticoagulant activity of the extracted oils was estimated by calculation of prothrombin time (PT) and clotting time (CT) according to (Pharmacopeia 1960) with some modifications (Hashem et al. 2018) using heparin as control. Hard-glass, test tubes were used. Each tube contained 0.8 mL of saline solution (0.9% w/v) or standard heparin solution (comprising 1 mg/tube) or test sample (100 µL /tube), and for blank, sample test was only added. To each tube prepared, 1.0 mL of human plasma and 0.2 mL of calcium chloride solution (2.0% w/v) were added. Time was immediately recorded and each tube was stoppered and the contents were mixed by inverting three times, in such a way that the entire inner surface of the tube was wet. The time required for clotting was determined.

### Fibrinolytic activity of the oil

The percentage of lysing a plasma clot was used to determine the fibrinolytic activity (Pharmacopeia 1960) by using hemoclar as a control. Sets of three hard glass test tubes were cleaned by immersion overnight in chromic acid. To each tube, 0.8 mL saline solution (0.9% w/v), 1.0 mL of prepared plasma and 0.2 mL calcium chloride (2.0% w/v) were added. After mixing, the tubes were placed in a water bath at 37 °C and when clotting was completed, 1.0 mL of either saline solution (0.9% w/v), hemoclar standard (2 mg/tube), or test sample (oil) solution (100 µl /tube) was added individually to the appropriate tube of blank, standard or sample, respectively. The percentage of lysis of plasma clot, at 37 °C for 24 h., was recorded and compared to that of standard “Hemoclar”. All samples were analyzed in triplicate.

### Yoghurt manufacture

Egyptian buffalo milk, containing 6.1% fat, 3.2% protein, and 15.8% total solids, was pasteurized at 85 °C for 15 min. Following pasteurization, the milk was divided into two equal portions. The first portion served as the control (C), with no additional mixed oils (MO) added. The second portion was supplemented with 25 µL of MO per liter of milk. Both portions were then inoculated with a commercial yoghurt starter culture, consisting of *Streptococcus thermophilus* and *Lactobacillus delbrueckii ssp. bulgaricus*, at a concentration of 3%. The inoculated samples were incubated at 42 °C until the pH reached 4.6. After incubation, the yoghurt was stored at 4 °C for 21 days.

### Yoghurt supernatant

The yoghurt supernatant was prepared following the method described by Abdel-Hamid et al. (2020). Briefly, approximately 50 g of yoghurt samples were centrifuged at 20,000 g for 30 min at 4 °C. The supernatants were then filtered through 0.45 μm cellulose acetate Whatman filter paper (Sigma, USA). The filtered supernatant was stored at − 20 °C until further analysis for phenolic content and antioxidant activity.

### Determination of total phenolic content

The total phenolic content (TPC) of the yoghurt samples was determined using a modified version of the Folin-Ciocalteu method as outlined by Meda et al. (2005). In brief, 500 µL of yoghurt extract was diluted with 500 µL of deionized water. A 100 µL aliquot of this diluted extract was then mixed with 2.8 mL of deionized water, 2 mL of 2% sodium carbonate (Na₂CO₃), and 0.1 mL of 50% Folin-Ciocalteu reagent. The mixture was incubated at room temperature for 30 min, after which the absorbance was measured at 750 nm using a spectrophotometer. Distilled water was used as a blank. Gallic acid (GA) served as the standard phenolic compound, with a standard curve prepared using seven concentrations ranging from 0 to 200 mg/L. The TPC was determined in triplicate for each sample, and the results were expressed as milligrams of Gallic acid equivalents (GAE) per gram of lyophilized powder, which was then converted to milligrams of GAE per gram of dry weight.

### Statistical data analysis

Statistical data analysis was performed using SPSS software to evaluate the significance of the results obtained from the various assays conducted in this study. Descriptive statistics, including means and standard deviations, were calculated for all measurements to summarize the data. For comparisons between groups, one-way analysis of variance (ANOVA) was employed, followed by post hoc tests (Tukey’s HSD) to identify significant differences among the means of different oil treatments. A significance level of *p* < 0.05 was established for all the statistical tests. The results were presented in tables and figures to facilitate interpretation and comparison, providing a comprehensive understanding of the potential health benefits associated with each oil tested.

## Results

### GC-MS analysis of oils and their mixture

The results of the GC-MS analysis of the six oils and their mixture, as presented in Table 1, revealed significant variations in fatty acid composition, which may contribute to their respective health benefits and functional properties. Notably, Black seed oil and Wheat germ oil exhibit high levels of linoleic acid (C18:2n6c), with values of 55.66% and 53.63%, respectively, indicating their potential as sources of essential fatty acids that support cardiovascular health. In contrast, Moringa oil stands out with an exceptionally high oleic acid (C18:1n9c) content of 74.26%, which is known for its beneficial effects on cholesterol levels and overall heart health.

Additionally, the presence of palmitic acid (C16:0) in all oils suggests a common characteristic that may influence their stability and shelf life (Goldschmidt and Byrdwell 2021). The mixture of oils shows a balanced profile, combining the beneficial properties of each oil, particularly in terms of unsaturated fatty acids, which are linked to anti-inflammatory effects and improved metabolic health (Runde et al. 2023). The varying levels of other fatty acids, such as stearic acid (C18:0) and linolenic acid (C18:3), further indicate the diverse applications these oils may have in functional foods and nutraceuticals. Overall, the GC-MS analysis provides valuable insights into the chemical composition of these oils, highlighting their potential health benefits and applications in the food and pharmaceutical industries (Mota et al. 2021; Runde et al. 2023).

**Table 1:** Fatty acid identification of GC-MS for the tested oil and their mixture

Fatty acids	BSO	WGO	CSO	CPO	MO	NO	Mixture oils
Myristic acid (C14:0)	0.26 ± 0.03	0.05 ± 0.01	ND	ND	0.62 ± 0.11	ND	0.19 ± 0.05
Palmitic acid (C16:0)	13.45 ± 1.21	13.43 ± 1.04	8.72 ± 0.57	10.56 ± 0.85	6.33 ± 0.72	14.32 ± 0.88	9.81 ± 0.28
Palmitoleic acid (C16:1)	ND	0.12 ± 0.06	0.52 ± 0.02	1.97 ± 0.37	1.66 ± 0.21	ND	0.58 ± 0.04
Stearic acid (C18:0)	2.62 ± 0.16	3.51 ± 0.15	4.54 ± 0.45	11.67 ± 1.04	4.14 ± 0.42	14.2 ± 1.02	2.33 ± 0.26
Oleic acid (C18:1n9c)	25.81 ± 1.34	24.03 ± 1.86	8.29 ± 0.72	37.73 ± 2.03	74.26 ± 2.16	57.26 ± 2.13	38.73 ± 1.62
Linoleic acid (C18:2n6c)	55.66 ± 1.19	53.63 ± 1.88	19.26 ± 0.65	33.72 ± 1.09	0.80 ± 0.13	12.07 ± 0.55	31.72 ± 1.17
Linolenic acid (C18:3)	ND	4.42 ± 0.27	56.15 ± 1.83	0.96 ± 0.22	0.06 ± 0.01	ND	14.74 ± 1.02
Arachidic acid (C20:0)	2.20 ± 0.11	0.35 ± 0.03	1.39 ± 0.15	1.73 ± 0.12	3.06 ± 0.14	2.15 ± 0.23	1.04 ± 0.15
11-eicosenoic acid (C20:1)	ND	0.46 ± 0.16	ND	0.39 ± 0.06	2.83 ± 0.19	ND	0.28 ± 0.05
Behenic acid (C22:0)	ND	ND	1.13 ± 0.15	1.27 ± 0.18	6.24 ± 0.27	ND	0.58 ± 0.06

ND = Not detectable. Black seed oil (BSO), Wheat germ oil (WGO), Chia seed oil (CSO), *Calotropis procera* oil (CPO), Moringa oil (MO), and Neem oil (NO). Values are expressed as Mean ± Standard Deviation (SD).

### Antimicrobial activity

The antibacterial activities of the six tested individual oils and the mixture against the six microorganisms are described in Table 2. The results show that the chosen oils exhibited differing levels of antimicrobial activity. A zone of inhibition larger than 10 mm in diameter was considered positive. Generally, most of the tested organisms were sensitive to many of the essential oils (*Calotorpis pracera*, Chia, Moringa, Neem, Black seed, and Wheat germ), and all of these showed antimicrobial activity. *Calotorpis pracera* showed maximum activity against the microorganisms tested. Mycostatin was used as positive control for *Candida albicans*, and *Aspergillus niger*. Mycostatin caused inhibition in growth by 68% for *Candida albicans* and 88% for *Aspergillus niger*. While Nystatin caused inhibition in growth by 59% and 90% for *Candida albicans* and *Aspergillus niger* respectively.

**Table 2:** Inhibition zones of different plant and seed oils against some pathogenic bacteria and fungi by well diffusion method (Size of well by mm)

Seed oil	Inhibition zone (mm)					
	Gram positive bacterial strains		Gram negative bacterial strains		Fungi and yeast	
	*Bacillus subtilus*	*Staphylococcus aureus*	*Escherichia coli*	*Pseudomonas aeruginosa*	*Candida albicans*	*Aspergillus niger*
*Calotorpis pracera*	30 ± 0.58^b^	32 ± 1.00^b^	33 ± 1.00^a^	20 ± 1.53^b^	33 ± 1.00^b^	-Ve
*Chia seed*	25 ± 1.53^cd^	24 ± 1.15^d^	30 ± 1.15^b^	22 ± 0.00^a^	23 ± 1.53^e^	20 ± 0.58^c^
*Moringa*	20 ± 0.00^e^	18 ± 0.53^e^	21 ± 0.00^d^	-Ve	22 ± 1.15^ef^	29 ± 0.00^a^
*Neem*	26 ± 1.15^c^	16 ± 0.58^f^	11 ± 0.00^f^	12 ± 0.00^e^	27 ± 1.15^d^	-Ve
*Black seed*	32 ± 0.58^a^	28 ± 1.53^c^	26 ± 1.00^cd^	14 ± 1.15^de^	31 ± 0.00^c^	13 ± 0.58^d^
*Wheat germ*	24 ± 1.53^d^	23 ± 1.00^de^	27 ± 1.53^c^	15 ± 1.53^d^	23 ± 1.00^e^	12 ± 0.00^f^
Mixture oil	31 ± 1.00^ab^	37 ± 0.00^a^	20 ± 0.58^de^	21 ± 1.53^ab^	39 ± 0.00^a^	25 ± 0.00^b^
Ampicillin	17 ± 0.00^f^	18 ± 1.53^e^	15 ± 0.00^e^	17 ± 0.58^c^	NA	NA

Size of well 9 mm, NA: is not applicable, –Ve: are negative results; Ampicillin and mycostatine were used as controls. Values presented as the mean ± SD (*n* = 3). In contrast to varying letters within the same row indicating a significant disparity between treatments, identical letters suggest no significant differences between treatments (*P* < 0.05).

### Minimum inhibitory concentration (MIC)

The minimum inhibitory concentration (MIC) for the six oils at a concentration of 100 mg/ml is shown in Table 3. *Calotorpis pracera* and *Black seed* oils with MIC activity values ranging from 15 to 50 µL, *Chia* seed oil with MIC activity values ranging from 20 to 30 µL, *Moringa* oil with MIC activity values ranging from 20 to 40 µL, *Neem* oil with MIC activity values ranging from 25 to 100 µL, and Wheat germ with MIC activity values ranging from 20 to 50 µL.

**Table 3:** Minimum inhibitory concentration (MIC µL) of individual oil and the mixture at a concentration of 100 mg/ml on tested microorganisms

Seed oil	Bacillus subtilus	Staphylococcus aureus	Escherichia coli	Pseudomonas aeruginosa	Candida albicans	Aspergillus niger
*Calotorpis pracera*	20 ± 0.00^c^	15 ± 1.15^e^	15 ± 1.53^d^	50 ± 1.00^b^	15 ± 1.00^c^	-Ve
*Chia seed*	25 ± 0.58^b^	25 ± 1.53^c^	20 ± 1.15^c^	30 ± 0.00^c^	25 ± 0.00^b^	30 ± 1.15^a^
*Moringa*	35 ± 1.53^a^	40 ± 0.00^b^	30 ± 0.00^b^	-Ve	30 ± 1.15^a^	25 ± 1.00^b^
*Neem*	25 ± 0.00^b^	75 ± 0.00^a^	100 ± 0.00^a^	100 ± 3.00^a^	25 ± 1.58^b^	-Ve
*Black seed*	20 ± 1.15^c^	20 ± 0.58^d^	20 ± 0.00^c^	50 ± 2.00^b^	15 ± 0.00^c^	50 ± 0.00^a^
*Wheat germ*	25 ± 0.00^b^	25 ± 1.15^c^	20 ± 1.53^c^	50 ± 2.00^b^	25 ± 1.00^b^	50 ± 1.00^a^
*The mixture*	1 ± 0.00^d^	1 ± 0.00^f^	2.5 ± 0.00^e^	2.5 ± 0.00^d^	2 ± 0.00^d^	1 ± 0.00^d^

–Ve: are negative results; Ampicillin and mycostatine were used as controls. Values presented as the mean ± SD (*n* = 3). In contrast to varying letters within the same row indicating a significant disparity between treatments, identical letters suggest no significant differences between treatments (*P* < 0.05).

### The prebiotic activity of oil

The prebiotic activity of the tested oils, as well as a mixture of these oils, was evaluated based on their effects on three probiotic strains of *Lactobacillus*: *Lactobacillus casei*,* Lactobacillus reuteri*,* and Lactobacillus helveticus.* The results, summarized in Table 4, indicate that the prebiotic effects varied from moderate to strong depending on the specific probiotic strain tested. Notably, the mixture of oils exhibited the strongest prebiotic activity, significantly enhancing the growth of all three Lactobacillus strains. In particular, Black seed oil demonstrated a robust prebiotic effect, with a prebiotic index (I) of 1.61 for *L. casei*, 0.66 for *L. reuteri*, and 0.48 for *L. helveticus*. In contrast, Calotropis procera oil showed the weakest prebiotic effect among all the oils tested, with an index of only 0.57 for *L. casei* and 0.28 for *L. reuteri*. The other oils, such as Wheat germ oil and Neem oil, displayed moderate prebiotic activity, with indices ranging from approximately 0.74 to 1.10 across the different probiotic strains. These results suggest that while certain oils can effectively promote the growth of beneficial bacteria, others may have limited prebiotic potential. Overall, these findings highlight the potential of specific oils, particularly when combined, to serve as effective prebiotics that support gut health by enhancing the growth of beneficial probiotics while inhibiting pathogenic bacteria like *E. coli*.

**Table 4:** Prebiotic activity of oil, and mixture on *Lactobacillus Sp*

Oil samples	L. casei (I)	L. reuteri (I)	L. helveticus (I)
Black seed oil	1.61 ± 0.05^b^	0.66 ± 0.12^d^	0.48 ± 0.17^g^
Wheat germ oil	1.10 ± 0.06^c^	0.90 ± 0.20^b^	0.80 ± 0.11^c^
Neem oil	1.00 ± 0.09^d^	0.82 ± 0.16^c^	0.74 ± 0.06^e^
Moringa oil	0.89 ± 0.03^e^	0.89 ± 0.08^bc^	0.78 ± 0.03^d^
*Calotropis procera* oil	0.57 ± 0.15^f^	0.28 ± 0.04^f^	0.56 ± 0.21^f^
Chia seed oil	1.12 ± 0.19^cd^	0.41 ± 0.25^e^	1.13 ± 0.11^b^
The mixture oil	2.48 + 0.21^a^	2.41 ± 0.12^a^	2.45 + 0.16^a^

I = prebiotic index = ratio of bacterial growth/pathogenic *E. coli* growth.

I = negative effect when equal to or less than 1.0.

I = positive effect when more than 1.0.

Values presented as the mean ± SD (*n* = 3). In contrast to varying letters within the same row indicating a significant disparity between treatments, identical letters suggest no significant differences between treatments (*P* < 0.05).

### Antioxidant activity

#### DPPH assay

**Fig. 1:**
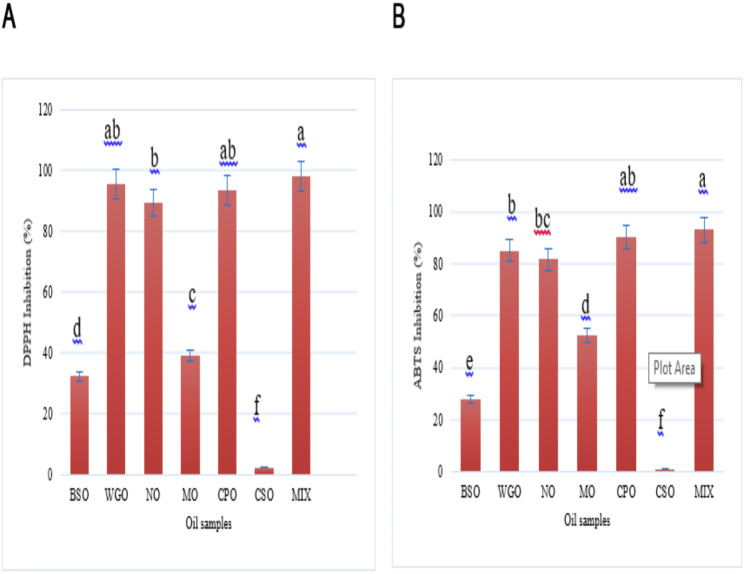
Antioxidant activity assay DPPH inhibition (**A**) and ABTS inhibition (**B**) of oils and their mixture. Black seed oil (BSO), Wheat germ oil (WGO), Neem oil (NO), Moringa oil (MO), *Calotropis procera* oil (CPO) and Chia seed oil (CSO). Error bars represent the standard deviation (± SD) (*n* = 3). Different letters assigned to values within the same samples denote statistical variances as per a two-way analysis of variance (*p < 0.05*)

The antioxidant activity of the tested oils and their mixture, as determined by the DPPH assay, revealed that Wheat germ oil exhibited the highest antioxidant capacity, with an activity of 95.76% ± 0.12. In contrast, Black seed oil demonstrated the lowest antioxidant effect among the oils tested, showing activity of only 32.38% ± 0.09, as mentioned in Fig. 1A. This indicates that while Wheat germ oil is highly effective at scavenging free radicals, Black seed oil has relatively limited antioxidant properties.

#### ABTS assay

The antioxidant activity of the tested oils and their mixture was also evaluated using the ABTS assay, which indicated that *Calotropis procera* oil had the strongest antioxidant activity among the oils tested, achieving an activity level of 90.56% ± 0.98. Conversely, Black seed oil again showed the weakest antioxidant effect, with an activity of only 27.89% ± 0.35, as shown in Fig. 1B. These results suggest that while *Calotropis procera* oil is effective in neutralizing free radicals, Black seed oil has limited efficacy in this regard.

### Anticoagulant activity of oil

**Fig. 2:**
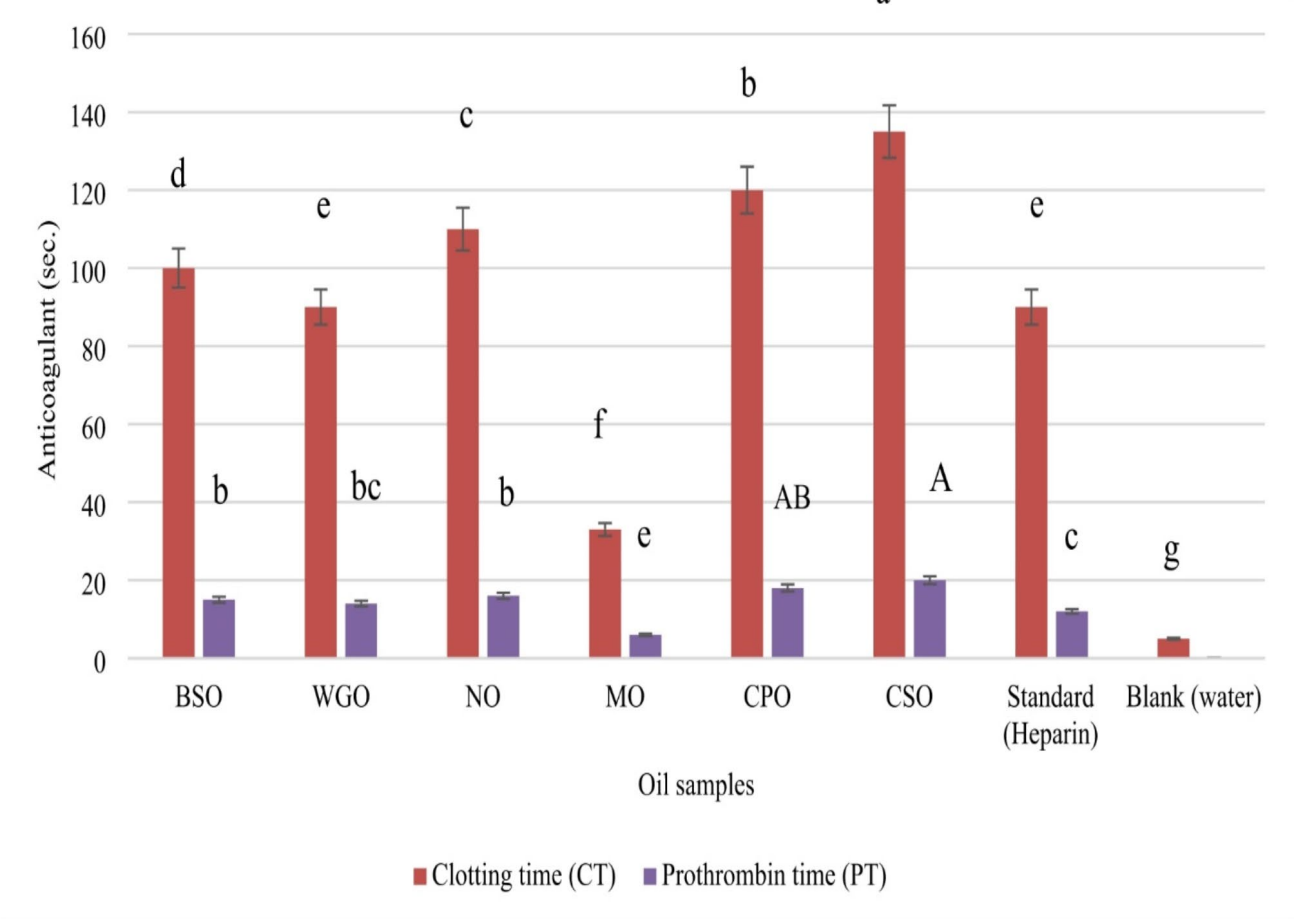
Anticoagulant activity of the tested oils. Black seed oil (BSO), Wheat germ oil (WGO), Neem oil (NO), Moringa oil (MO), *Calotropis procera* oil (CPO) and Chia seed oil (CSO). Error bars represent the standard deviation (± SD) (*n* = 3). Different letters assigned to values within the same samples denote statistical variances as per a two-way analysis of variance (*p* < 0.05)

The anticoagulant activity of the tested oils, compared to the standard anticoagulant heparin, is shown in Fig. 2. Among the oils evaluated, *Chia seed oil* demonstrated the strongest anticoagulant effect, with a clotting time (CT) of 135 s. and a prothrombin time (PT) of 20 s. Following closely was *Calotropis procera* oil, which exhibited a CT of 120 s. and a PT of 18 s. *Neem oil* also showed notable anticoagulant properties, with a CT of 110 s. and a PT of 16 s. In contrast, *Moringa seed oil* displayed the weakest anticoagulant activity, with a significantly lower CT of just 33 s. and a PT of 6s.

### Fibrinolytic activity of the oil

**Fig. 3:**
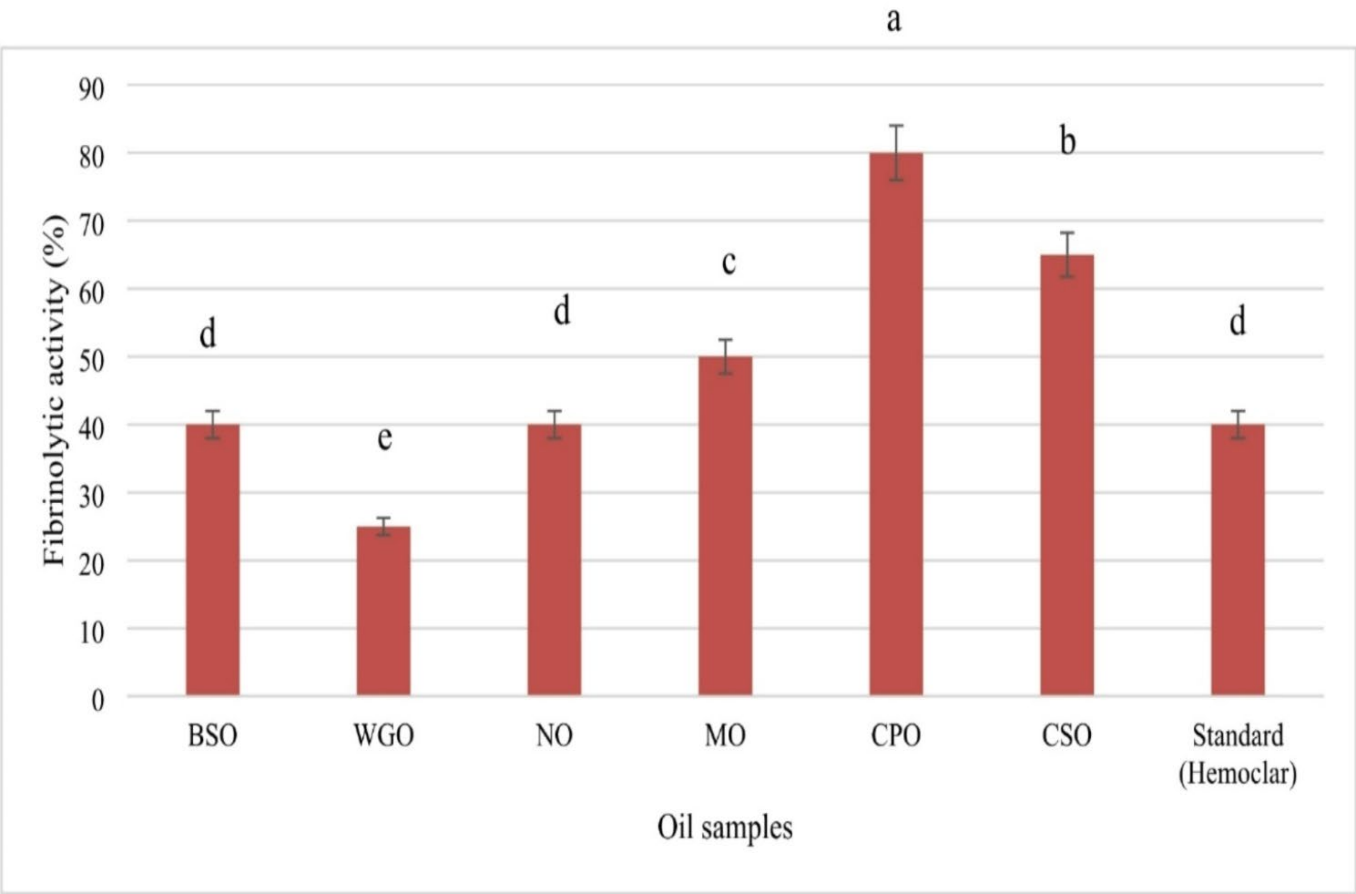
Fibrinolytic activity of the tested oils. Black seed oil (BSO), Wheat germ oil (WGO), Neem oil (NO), Moringa oil (MO), *Calotropis procera* oil (CPO) and Chia seed oil (CSO). Error bars represent the standard deviation (± SD) (*n* = 3). Different letters assigned to values within the same samples denote statistical variances as per a two-way analysis of variance (*p* < 0.05)

The fibrinolytic activity of the tested oils, compared to the control standard Hemoclar, is presented in Fig. 3. Both *Black seed oil* and *Neem oil* demonstrated fibrinolytic activity equivalent to that of the control, each showing an activity of 40%. In contrast, *Calotropis procera* oil exhibited the highest fibrinolytic activity among the oils tested, with a remarkable activity level of 80%. Following this, *Chia seed oil* displayed a substantial fibrinolytic effect of 65%, while Moringa oil showed a moderate activity of 50%. The weakest fibrinolytic activity was observed in Wheat germ oil, which recorded an activity of only 25%.

### Total phenolic content and antioxidant activity of the prepared yoghurt samples

**Fig. 4:**
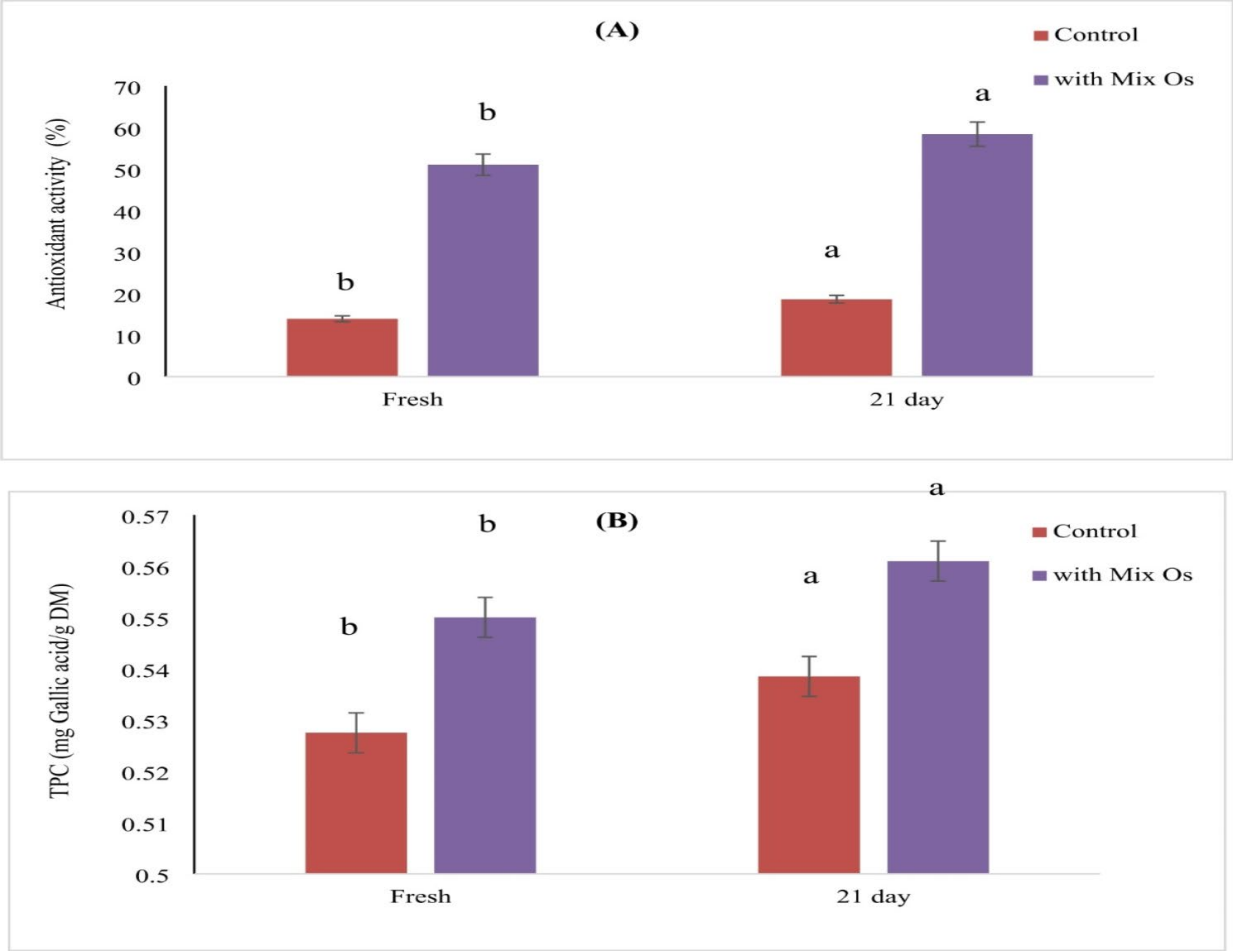
The antioxidant activity (**A**) and total phenolic content (TPC) (**B**) of yoghurt samples with/out MO. Error bars represent the standard deviation (± SD) (*n* = 3). Different letters assigned to values within the same samples denote statistical variances as per a two-way analysis of variance (*p* < 0.05)

The results for the antioxidant activity (AA) and total phenolic content (TPC) of yoghurt samples, both with and without the addition of mixed oils (MO), are shown in Fig. 4A and B. On day 0 of storage, the antioxidant activity of the control yoghurt was 13.93 ± 2.52%, while that of the yoghurt fortified with MO was significantly higher at 51.04 ± 2.67%. Similarly, the TPC was 0.528 ± 0.005 mg Gallic acid/g dry matter (DM) in the control and 0.550 ± 0.003 mg Gallic acid/g DM in the fortified yoghurt.

## Discussion

The results of this study demonstrate that the selected oils exhibited varying levels of antimicrobial activity. Overall, most of the tested organisms were sensitive to the oils, including *Calotropis procera*, *Chia*, *Moringa*, *Neem*, *Black seed*, and *Wheat germ*, all of which displayed moderate to strong antimicrobial and antioxidant properties. However, most of the oils tested showed limited or weak prebiotic effects on the probiotic strains examined. In contrast, the mixture of oils exhibited superior antimicrobial, antioxidant, and prebiotic activities compared to each oil. Among the oils tested, *Chia seed oil* exhibited the strongest anticoagulant activity, ranking second only to *Calotropis procera* oil in terms of fibrinolytic activity. This finding aligns with Oliva et al. (2021), who noted that chia seed supplementation serves as an effective functional food and dietary strategy for preventing or reversing atherothrombotic cardiovascular disorders and liver fibrosis. Their research demonstrated that feeding rats a chia seed-enriched diet for three months reversed several diet-induced diseases, including dyslipidemia, visceral adiposity, and insulin resistance. Additionally, they observed normalization of platelet counts, coagulation parameters, and plasma fibrinogen levels in these animals. Our findings are also consistent with Ozón et al. (2022), who reported antioxidant, anticoagulant, and antithrombotic activities of chia seeds and their by-products against both intrinsic and extrinsic coagulation pathways. However, it is noteworthy that Ozón et al. (2022) did not detect antioxidant activity in chia seed oil in their study. *Chia seed oil* demonstrated significant antimicrobial activity, corroborated by other studies (Elshafie et al. 2018; Parker et al., 2018). Furthermore, various reports have documented the antifungal and antibacterial properties of different chia seed products, such as mucilage (Muñoz-Tébar et al. 2022; Cacciatore et al. 2022). Our results are comparable to those of Knez et al. (2019), who highlighted chia’s substantial antimicrobial capacity, likely attributed to its high linolenic acid (C18:3) content (56.15%). However, *Chia seed oil* exhibited a weak prebiotic effect on two strains of *Lactobacillus*, *L. casei* and *L. helveticus*, while showing no effect on *L. reuteri*.

Among all six oils tested, *Calotropis procera* oil demonstrated maximum antioxidant and antimicrobial activity against the tested bacterial strains. This efficacy may be linked to its high oleic acid (C18:1) and linoleic acid (C18:2) content (37.73% and 33.72%, respectively), which are well-known for their antibacterial properties (Dilika et al. 2000; Huang et al. 2010; Petropoulos et al. 2021) as well as their antioxidant activity in medicinal plants and seeds (Saini et al. 2017; Türkekul et al. 2017; Song et al. 2021). However, it was ineffective against the tested fungus (*Aspergillus niger*). Our findings align with Amini et al. (2021), who reported significant antibacterial activity from *C. procera* extracts, as well as with Yesmin et al. (2008), who documented its antioxidant activity through DPPH radical scavenging assays. The present study’s findings regarding *C. procera* are consistent with research conducted by Al-Rowaily et al. (2020a, b), which indicated that essential oil from *C. procera* exhibited antibacterial activity against various bacteria and fungi. Conversely, this oil showed no prebiotic activity on the tested *Lactobacillus* strains in our study. Notably, *Calotropis procera* oil proved to be a strong anticoagulant, second only to *Chia seed oil*. It also exhibited greater fibrinolytic activity than *Chia seed oil*. This is supported by Omar et al. (2023), who reported concentration-dependent anticoagulant activity from various extracts of *Calotropis procera*, suggesting inhibitory effects on clotting factors within both intrinsic and extrinsic pathways. When compared to the positive control heparin, *Calotropis procera* oils demonstrated relatively good prothrombin time (PT) values (18 ± 0.15 s) and longer clotting times (120 s.).

Our findings revealed that *Calotropis procera* oil exhibited the most significant antimicrobial activity against the tested bacterial strains, followed by Chia seed oil. This strong antimicrobial effect may be attributed to the presence of specific bioactive compounds in these oils. *Calotropis procera* oil contains various cardenolides, alkaloids, and terpenoids (Singh et al. 2024). These compounds are known for their ability to disrupt bacterial cell membranes, inhibit protein synthesis, and interfere with essential metabolic processes (Ahmad Nejhad et al. 2023). Specifically, cardenolides can inhibit the Na+/K+-ATPase pump in bacterial cell membranes, leading to cell death (Yang et al. 2018). The higher concentration of these potent compounds in *Calotropis procera* oil likely contributes to its superior antimicrobial efficacy compared to other oils tested.

Chia seed oil, while less potent than *Calotropis procera* oil, also demonstrated significant antimicrobial activity. This activity may be due to its high content of α-linolenic acid (ALA), a polyunsaturated fatty acid, and other phenolic compounds such as chlorogenic acid, caffeic acid, and myricetin (Mettwally et al. 2022a, b). ALA has been shown to disrupt bacterial membrane integrity and inhibit the growth of various pathogens (Casillas-Vargas et al. 2021). Furthermore, the phenolic compounds present in chia seed oil can inhibit bacterial enzymes involved in cell wall synthesis and energy production, leading to growth inhibition (Abdel-Aty et al. 2021). The synergistic effect of ALA and phenolic compounds likely contributes to the observed antimicrobial activity of chia seed oil.

The antioxidant activity was highest in *Calotropis procera* and Wheat germ oils, suggesting the presence of potent radical scavenging compounds. *Calotropis procera* oil is known to contain various phenolic compounds, flavonoids, and terpenoids (Ahmad Nejhad et al. 2023; El-Hadad et al. 2024). Flavonoids act as antioxidants by donating electrons or hydrogen atoms to stabilize free radicals, thereby preventing oxidative damage to cellular components (Panche et al. 2016). Terpenoids also contribute to antioxidant activity through similar mechanisms and by enhancing the activity of endogenous antioxidant enzymes (Kobayashi et al. 2025). The high antioxidant activity of *Calotropis procera* oil can therefore be attributed to the combined effect of these various antioxidant compounds.

Wheat germ oil is a rich source of vitamin E (tocopherols), particularly alpha-tocopherol, and also contains other antioxidants such as carotenoids and ferulic acid (El-Hadad et al. 2024). Alpha-tocopherol is a well-known chain-breaking antioxidant that protects cell membranes from lipid peroxidation by scavenging lipid peroxyl radicals (Burton et al. 1985). Carotenoids and ferulic acid also contribute to the overall antioxidant capacity of wheat germ oil by neutralizing free radicals and reducing oxidative stress (Kobayashi et al. 2025). The high concentrations of these antioxidants in wheat germ oil likely explain its strong antioxidant activity observed in this study.”

Meanwhile, *Moringa oil* displayed good antimicrobial activity against all the tested pathogens except for *Pseudomonas aeruginosa*. This observation is consistent with findings from Lalas et al. (2012) and Sharma et al. (2019), which recognized the antimicrobial properties of *Moringa* oils. Chuang et al. (2007) also reported antifungal properties associated with Moringa essential oil. The strong antimicrobial activity of *Moringa oil* can be attributed to its high oleic acid content (74.26%), a fatty acid recognized for its antimicrobial properties (Rahdar et al. 2020; Ghavam et al. 2021, 2022). However, both its antioxidant and anticoagulant activities were found to be lower than those of other tested oils. De Andrade Luz et al. (2012) identified carbohydrate recognition proteins known as lectins that act as anticoagulant proteins affecting in vitro blood coagulation parameters by interacting through a carbohydrate recognition domain. Previous studies have reported fibrinolytic activities associated with aqueous extracts of *Moringa oleifera* leaves and roots (Satish et al. 2012). Interestingly, no prebiotic effect was observed from Moringa oil on the tested Lactobacillus strains in this study. This finding contrasts with Wang et al. (2019), who suggested that polysaccharides derived from *Moringa oleifera* leaves might serve as promising prebiotics with health benefits when tested in vivo on ICR mice. The discrepancy may arise from differences in the parts of *Moringa oleifera* that were tested; our study focused on seed oil rather than leaf powder. Furthermore, the prebiotic tests conducted here were performed in vitro rather than in vivo. *Neem oil* demonstrated good antimicrobial activity but failed to affect *Aspergillus niger*. This observation aligns with findings from Elavarasu et al. (2012) and SaiRam et al. (2000), who reported high antimicrobial properties associated with neem oil.

Additionally, Blum et al. (2007) found that neem oil extract exhibited substantial bactericidal effects against *Helicobacter pylori*, while Gupta and Bhat (2016) noted its antibacterial effects against methicillin-resistant *Staphylococcus aureus* (MRSA). Our results indicate that neem oil possesses strong antioxidant and anticoagulant activities; however, its fibrinolytic activity was found to be weak. These findings are supported by Rinaldi et al. (2017), who recorded significant antioxidant activity from neem oil nanoemulsions using DPPH and ABTS assays, as well as by Sani et al. (2022), who documented higher free radical scavenging activities for neem seed oil compared to flower extracts. Furthermore, Nahak and Sahu (2011) indicated that neem seed oil contains high levels of total phenolic compounds responsible for inhibiting DPPH radicals effectively. They also noted potential health benefits for incorporating neem flower and seed oils into human diets as healthy supplements for both diabetics and the general population due to their antioxidant potential for use in pharmaceuticals. The anticoagulant properties of neem oil have been previously reported by Mani et al. (2018), highlighting its applications in wound healing processes as well. On the other hand, neem seed oil did not exhibit any prebiotic activity on the tested Lactobacillus strains in this study; this finding contradicts results reported by Rehman et al. (2023), where neem herbal extract positively influenced intestinal microbial populations in broiler chicks when incorporated into their diet by increasing Lactobacillus content while decreasing *E. coli* levels.

The observed enhanced prebiotic activity of the oil mixture compared to individual oils suggests a synergistic effect in promoting the growth of beneficial bacteria. The mixture provides a diverse array of fatty acids and bioactive compounds that can selectively stimulate the growth of *Lactobacillus* strains. For example, specific fatty acids in chia seed oil and moringa oil may serve as preferential carbon sources for *Lactobacillus* species, while other compounds such as phenolic acids in black seed oil, may enhance their metabolic activity (Zeng et al. 2024). Additionally, the combination of different oils may create a more favorable microenvironment in the gut, promoting the colonization and growth of beneficial bacteria while inhibiting the growth of pathogens. Further studies are needed to identify the specific components responsible for the observed prebiotic activity and to elucidate the exact mechanisms by which they promote the growth of *Lactobacillus* strains.

*Black seed oil* showed a moderate prebiotic effect on *Lactobacillus casei* but had no impact on the other two Lactobacillus strains tested. These findings are consistent with reports from Kooti et al. (2016) and Mohammed et al. (2019), which highlighted the antimicrobial properties of black seed oil. Furthermore, Salman et al. (2016) demonstrated that black seed oil possesses antibacterial effects against multidrug-resistant bacteria; this antimicrobial capacity is attributed to its high linoleic acid (C18:2n6c) and oleic acid (C18:1n9c) content active components previously documented in black seed oils (Rahim et al. 2022; Joujou et al. 2024). In our study, the linoleic acid content in black seed oils was significantly higher than that found in all other tested oils at 55.66%. Our results align with Tiji et al.‘s findings from 2021 that linked the antibacterial effects of Moroccan *Nigella sativa* seed extracts to fatty acids such as linoleic acid and palmitic acid. Notably, black seed oil exhibited the weakest antioxidant activity among all six oils tested, along with weak fibrinolytic activity despite its strong anticoagulant properties. These observations are consistent with Yusof et al., (2017) regarding *Nigella sativa* seed extract’s potent anticoagulant effects Al-Jishi and Hozaifa, (2003). Additionally, indicated strong anticoagulant effects associated with *Nigella sativa* when administered to adult male albino rats at varying doses over four weeks. Other recent studies have reported the hematological effect of *Nigella sativa* (Benjamin, 2025; Chad 2025; Ozsan et al. 2024). Awad et al. (2005) suggest a concentration-dependent role for black seed oil in modulating fibrinolysis/thrombus formation through influencing endothelial cell fibrinolytic potential. Conversely, Awad et al. (2005) earlier work indicated that black seed oil decreased fibrinolytic potential in vitro within human fibrosarcoma cell lines (*HT1080*), suggesting possible mechanisms for inhibiting local tumour metastasis.

Lastly, wheat germ oil demonstrated the lowest fibrinolytic activity among all tested oils while exhibiting moderate anticoagulant effects alongside strong antioxidant properties against all microorganisms evaluated consistent with Kim et al.‘s findings from 2010 regarding wheat germ extract’s antimicrobial properties. GC-MS analysis revealed relatively high contents of oleic acid and linoleic acid at 24.03% and 53.63%, respectively—both recognized for their antibacterial activities, as previously mentioned. Moreover, Mahmoud et al.‘s research from 2015 noted strong antibacterial effects associated with defatted wheat germ extracts. Wheat germ oil emerged as an effective stimulant for promoting growth among prebiotic bacteria such as *L. reuteri* while showing no impact on the other two Lactobacillus species evaluated. In summary, regarding anticoagulant activities among these oils, both chia seed oil, followed closely by *calotropis procera*, exhibited robust effects while moringa oil displayed minimal impact overall; *calotropis procera* also showcased superior fibrinolytic capabilities, followed by chia seed oil, whereas wheat germ demonstrated weaker activities across these parameters highlighting their potential applications across various health-related fields.

The observed increase in both antioxidant activity (AA) and total phenolic content (TPC) in the fortified yoghurt can be attributed to the bioactive peptides naturally present in milk (Korhonen & Pihlanto, 2020), as well as the incorporation of a multifunctional oil (MO) blend. This blend, comprising *Calotropis procera* oil, chia seed oil, moringa oil, neem oil, black seed oil, and wheat germ oil, provides a rich source of antioxidants and phenolic compounds, contributing synergistically to enhancing the yoghurt’s overall antioxidant capacity (Usmani et al., 2023; Ixtaina et al., 2022; Rani et al., 2018). Unlike previous studies that have focused on individual oils, this research explores the combined effect of a diverse oil blend, potentially offering a more effective strategy for enhancing functional properties in dairy products (Sharma et al. 2023).

Each oil within the blend possesses a unique phenolic profile. *Calotropis procera* oil, despite its relatively low phenolic content, contains phenolic acids such as P-coumaric acid and flavonoids like catechin and rutin, which have demonstrated significant antioxidant potential (Usmani et al., 2023). Chia seed oil is particularly rich in chlorogenic acid, caffeic acid, myricetin, quercetin, and kaempferol, contributing to its strong antioxidant activity (Ixtaina et al. 2023). Moringa oil enhances the mixture with Gallic acid, chlorogenic acid, ellagic acid, and flavonoids, which are known for their free radical scavenging properties (Rani et al., 2018). Neem oil contributes additional antioxidant compounds, including nimbin, quercetin, and Gallic acid ( Ni Putu Ratna et al., 2024). Black seed oil, recognized for its health-promoting properties, is rich in thymoquinone, carvacrol, t-anethole, and 4-terpineol, which significantly enhance antioxidant activity (Soleimanifar et al. 2019). Lastly, wheat germ oil further strengthens the blend’s antioxidant capacity with ferulic acid, vanillic acid, p-hydroxybenzoic acid, and flavonoids such as apigenin and luteolin ( Mohammed et al. 2023).

The combination of these oils results in an amplified antioxidant effect due to the complementary actions of their diverse phenolic compounds. The synergistic interactions among these bioactive components effectively scavenge free radicals, mitigate oxidative stress, and provide enhanced cellular protection. Furthermore, phenolic compounds are known to modulate enzymatic antioxidant systems. The presence of this oil blend may have contributed to the upregulation of endogenous antioxidant enzymes such as catalase and superoxide dismutase during storage, further enhancing the stability of antioxidant activity.

Over the storage period, a progressive increase in both AA and TPC was observed, particularly by the 21st day (Fig. 2). This increase may be attributed not only to the activation of enzymatic antioxidants but also to potential interactions between phenolic compounds and milk proteins, which could enhance their bioavailability (Stobiecka et al., 2022). A similar trend was reported by Kang et al. (2018), who found that the AA of yogurt increased significantly with the addition of red or green pepper juice, establishing a direct correlation between AA levels and TPC (Kang et al. 2018).

From a functional perspective, the fortification of yoghurt with this oil blend not only enhances its nutritional value but may also influence sensory attributes such as texture and flavor (Shiby and Mishra 2013). While previous studies have reported that high levels of phenolic compounds can lead to bitterness or astringency (Buitimea-Cantúa et al. 2018), the balanced formulation of this oil blend likely minimizes undesirable effects while maximizing health benefits. The incorporation of plant-based bioactive compounds has been shown to improve not only antioxidant potential but also extend the shelf-life of dairy products by reducing lipid oxidation and microbial spoilage (Ali et al. 2022). Future research should focus on consumer acceptability studies and the potential impact of this oil blend on gut microbiota, as phenolic-rich ingredients have been shown to modulate intestinal microbial composition and promote the growth of beneficial bacteria (Loo et al. 2020), further validating its functional food applications.

This study highlights the diverse health benefits of various seed oils, including *Calotropis procera*, *Chia*, *Moringa*, *Neem*, *Black seed*, and *Wheat germ*, emphasizing their potential applications in functional foods. The oils demonstrated varying levels of antimicrobial, antioxidant, anticoagulant, and prebiotic activities, with *Chia seed oil* exhibiting the strongest anticoagulant properties and significant antimicrobial effects. Conversely, *Calotropis procera* oil showed the highest antioxidant and antimicrobial activities, making it a promising candidate for health enhancement. While most oils displayed weak prebiotic effects on tested probiotic strains, the mixture of oils demonstrated superior activity across all evaluated parameters. Additionally, the findings indicate that *Moringa oil* possesses notable antimicrobial properties but limited anticoagulant effects, while *Neem oil* exhibited strong antioxidant and antimicrobial activities. Notably, *Black seed oil* showed robust anticoagulant properties despite its weaker antioxidant and fibrinolytic activities. The study underscores the potential of these seed oils as functional food ingredients that can contribute to improved health outcomes through their bioactive compounds. Future research should further explore the mechanisms underlying these effects and investigate the practical applications of these oils in dietary interventions for enhanced health benefits.

## Conclusion

In conclusion, our study demonstrates the significant potential of *Calotropis procera*, chia seed, moringa, neem, black seed, and wheat germ oils, particularly in combination, as functional ingredients in both food products and nutraceuticals. The observed antimicrobial activity, especially against common foodborne pathogens, suggests their utility as natural preservatives in yoghurts, cheeses, and other dairy products, reducing reliance on synthetic additives. Furthermore, the potent antioxidant properties of these oils support their incorporation into nutraceutical formulations aimed at combating oxidative stress and promoting overall health. The prebiotic potential, particularly of the oil mixture, indicates a valuable role in enhancing gut health when incorporated into functional foods. Future research should focus on optimizing oil concentrations and conducting in vivo studies to validate these health benefits, paving the way for their wider adoption in innovative food and nutraceutical applications.

## Data Availability

No datasets were generated or analysed during the current study.
